# Is the Relationship between Common Mental Disorder and Adiposity Bidirectional? Prospective Analyses of a UK General Population-Based Study

**DOI:** 10.1371/journal.pone.0119970

**Published:** 2015-05-18

**Authors:** Léopold K. Fezeu, David G. Batty, Catharine R. Gale, Mika Kivimaki, Serge Hercberg, Sebastien Czernichow

**Affiliations:** 1 Université Paris 13, Sorbonne Paris Cité - Equipe de Recherche en Epidémiologie Nutritionnelle (EREN), Centre d’Epidémiologie et Biostatistiques (EPIBIOS), Inserm, Inra, Cnam, Université Paris 5, Université Paris 7, Bobigny, France; 2 Medical Research Council Epidemiology Resource Centre, University of Southampton, Southampton, United Kingdom; 3 Department of Epidemiology & Public Health, University College London, London, United Kingdom; 4 Medical Research Council Social and Public Health Sciences Unit, Glasgow, United Kingdom; 5 Finnish Institute of Occupational Health, Helsinki, Finland; 6 Département de Santé Publique, Hôpital Avicenne (AP-HP), Bobigny, France; 7 Department of Nutrition, Ambroise Paré Hospital (AP-HP); University of Versailles Saint Quentin en Yvelines, Boulogne-Billancourt, France; 8 INSERM, UMS011 Population-based cohort unit, Villejuif, France; San Francisco, UNITED STATES

## Abstract

The direction of the association between mental health and adiposity is poorly understood. Our objective was to empirically examine this link in a UK study. This is a prospective cohort study of 3 388 people (men) aged ≥ 18 years at study induction who participated in both the UK Health and Lifestyle Survey at baseline (HALS-1, 1984/1985) and the re-survey (HALS-2, 1991/1992). At both survey examinations, body mass index, waist circumference and self-reported common mental disorder (the 30-item General Health Questionnaire, GHQ) were measured. Logistic regression models were used to compute odds ratios (OR) and accompanying 95% confidence intervals (CI) for the associations between (1) baseline common mental disorder (QHQ score > 4) and subsequent general and abdominal obesity and (2) baseline general and abdominal obesity and re-survey common mental disorders. After controlling for a range of covariates, participants with common mental disorder at baseline experienced greater odds of subsequently becoming overweight (women, OR: 1.30, 1.03 – 1.64; men, 1.05, 0.81 – 1.38) and obese (women, 1.26, 0.82 – 1.94; men, OR: 2.10, 1.23 – 3.55) than those who were free of common mental disorder. Similarly, having baseline common mental health disorder was also related to a greater risk of developing moderate (1.57, 1.21 – 2.04) and severe (1.48, 1.09 – 2.01) abdominal obesity (women only). Baseline general or abdominal obesity was not associated with the risk of future common mental disorder. These findings of the present study suggest that the direction of association between common mental disorders and adiposity is from common mental disorder to increased future risk of adiposity as opposed to the converse.

## Introduction

Common mental disorder—comprising anxiety and depression—and obesity exact a considerable public health burden.[1,2] The secular rise in the prevalence of both in recent years has, in part, prompted speculation about their inter-relationship. In observational studies, there is growing evidence to suggest that people with common mental disorders have a greater risk of obesity than those who are apparently free of such mental health problems, although these findings are not entirely concordant. [3–5]There are also some studies that have explored the converse hypothesis: obesity as a risk factor for future mental disorders. Again, the results from these studies have been contradictory, with evidence of positive,[6,7] null,[8,9] and even an inverse association.[10]

Several mechanisms may explain these apparent bidirectional associations. These may be context specific and include attitudes towards body size and mental ill health in the population under study.[11] For example, in societies where obesity is stigmatised, being overweight or obese may lead to increased risk of anxiety and depression, whereas the reverse effect may be seen in communities where a large body size is prized. Additionally, commonly used treatments for depression have known side effects that result in weight gain.[12,13] Given the public health importance of increasing rates of both obesity and common mental disorder, understanding the nature of the association between these two conditions is crucial as it could inform prevention and treatment. This is particularly true in Western societies as the United Kingdom, where the prevalence of obesity almost doubled in the last three decades.[14,15]

Prospective studies with repeated measurements of both common mental disorder and different forms of obesity, such as general and abdominal adiposity, offer an important opportunity for an in-depth analysis of the inter-relationship between obesity and mental health. However, such studies are scarce.[16,17] The Health and Lifestyle Survey, which is a representative sample of the UK population, had an assessment of adiposity and mental health both at baseline and re-survey seven years later. These data afford us the opportunity to examine the potential for a prospective reciprocal association between common mental disorder and general or abdominal obesity.

## Methods

### Baseline examination (HALS-1)

In 1984/1985, the Health and Lifestyle Survey (HALS-1), was conducted in a random sample of the Population of England, Scotland and Wales aged 18 and over.[18,19] This study was designed as a unique attempt to describe self-reported health, attitudes and beliefs about causes of disease in relation to measurements of health and lifestyle in adults. A total of 12 672 addresses were selected from the electoral rolls of 198 randomly selected constituencies. After excluding empty or demolished dwellings and potential study members in hospital or living in residential accommodation, a total of 9 003 men and women were interviewed. The socio-economic profile of this sample compares favourably with UK census data based on selected variables.[20] Ethical approvals were obtained from the British Medical Association ethical committee.[18] Each study participant gave its written informed consent.

The baseline fieldwork consisted of three phases. A face-to-face interview was carried out to collect details on marital status, employment history, smoking habits, alcohol consumption, physical activity and long-standing illness or disability. Data on occupation were used to derive occupational social class in accordance with the scheme of the UK registrar General. Interviewees then had their height, weight and waist circumference measured by a trained nurse. Finally, these participants were requested to complete and mail a self-completion questionnaire assessing personality and psychiatric status, including the 30-item General Health Questionnaire (GHQ-30).[21]

### Re-survey (HALS-2)

**Fig 1:**
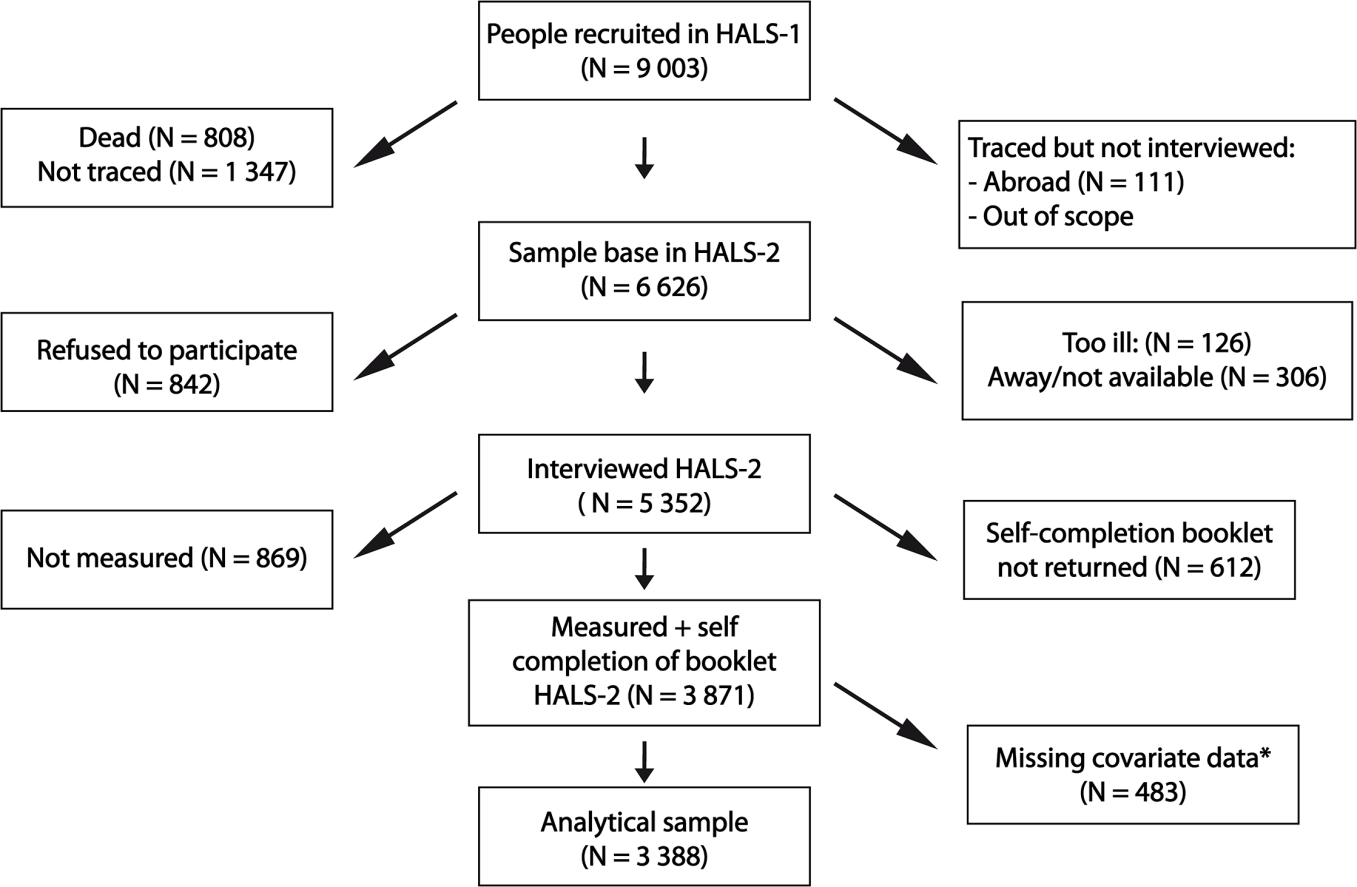
Participants selection flow in the HALS. *These subjects with missing data for any of the following covariates: age, general health questionnaire, marital status, occupational social class, alcohol consumption, smoking status, height, weight and waist circumference.

In 1991, seven years after the first survey, a follow-up study (re-survey) was carried out in order to identify changes that occurred in health and lifestyle among the original respondents.[18] Fig 1 shows the response at tracing and each level of interview. Of the original 9 003 participants of the baseline survey, a total of 6 626 respondents were traced, alive at the time of the re-survey and 81% (5 352) were successfully re-interviewed. From these, 869 participants were not measured for anthropometric variables and 612 participants did not return the GHQ questionnaire. The response proportion was higher in the younger age groups, and lowest among those over 80 years of age. A higher proportion of interviews were achieved in non-manual than manual occupational social classes. The response proportion for those undergoing the measurements by the nurse was 94.2% of those who had been measured at the baseline survey. The proportion of those returning the self-completion questionnaire that also returned it at the baseline survey was 84.1%. The analytical sample consisted of 3,388 participants with complete data on obesity and mental health at baseline and re-survey, and the covariates. All the data used for the present analyses are available upon request at http://www.esds.ac.uk/findingData/halsTitles.asp.

### Obesity definitions

Body mass index (BMI) was used to characterize general obesity according to the World Health Organization definition:[22] normal weight (BMI < 25 kg/m²), overweight (BMI between 25.0 and 29.9 kg/m²) or obesity (BMI > 30 kg/m²). Waist circumference was used to classify study participants as having a normal abdominal circumference (WC < 80 cm for women and 94 cm for men), a moderate (WC between 80 and 88 cm for women or 94 and 102 cm for men) or a severe (WC > 88/102 cm for women and men, respectively) abdominal obesity.[23]

### Mental disorders definitions

The GHQ-30, which provides an indication of common mental disorder, is a widely utilized measure of common mental disorder in population-based studies.[24] It consists of items relating to anxiety, depression, social dysfunction, and loss of confidence. Interpretation of the answers is based on a four points response scale scored using a bimodal method (symptom present: 'not at all' = 0, 'same as usual' = 0, 'rather more than usual' = 0 and 'much more than usual' = 1). We employed a cut off score of > 4 to denote common mental disorder.[25] This definition has been validated against standardised psychiatric interviews and has been strongly associated with various psychological disorders such as depression and anxiety.[25]

### Statistical analyses

Results are expressed as means (standard deviations, sd) or median (25th— 75th percentiles) for continuous variables and percentages for categorical variables. Gender specific logistic regression models were used, with adjustments for baseline age (model 1) then for baseline age, marital status, socio-economic status (occupational social class), alcohol consumption, tobacco smoking and physical activity levels (model 2). In order to examine the direction of the association we: (i) excluded subjects with baseline obesity in analyses of the influence of baseline common mental disorder on the onset of obesity at re-survey and (ii) excluded subjects with common mental disorder at baseline in analyses of the influence of adiposity on the onset of common mental disorder at re-survey. There was no interaction between age classes (<35, 35–54, > 55 years), for BMI or WC categories (all p>0.13, in men and women) in relation to psychological distress.

Gender specific simple and multinomial logistic regression models were used to study global obesity or abdominal obesity as a risk factor for the onset of common mental disorder at the re-survey study. Two definitions were used to characterize exposition to obesity. For general obesity, these were baseline normal weight versus overweight or obesity, and subjects with BMI < 30 kg/m² at baseline and re-survey versus subjects having a BMI ≥ 30 kg/m² at either or both studies. For abdominal obesity, these were baseline normal WC versus moderate or severe abdominal obesity, and subjects with WC < 88 cm (women) 102 cm (men) at baseline and re-survey versus subjects having a WC > 88 cm (women) 102 cm (men) at either or both studies.

To test the robustness of our results, statistical analyses were performed with BMI, WC and GHQ score as continuous variables, using linear regression models. For the two models adjusted described above, the prediction of 1) changes (second minus first survey) in BMI and WC by baseline GHQ score in two categories and 2) changes in GHQ score by baseline BMI and WC were computed. All statistical analyses were performed using Stata 10.1.

## Results

Compared to participants excluded from the analyses (n = 3375), there was no statistically significant difference for gender (p = 0.42), alcohol consumption (p = 0.39) and BMI (p = 0.64). However, excluded participants were one year older (p = 0.002), smoked more and had a one centimetre higher waist circumference (S1 Table).

Characteristics of the study population are shown in Table 1. At baseline, men had significantly higher BMI (p < 0.001) and waist circumference (p < 0.001), but the prevalences of global obesity (p < 0.009) and severe abdominal obesity (p < 0.065) were higher in women. As expected, common mental disorder was more frequent in women than in men (p < 0.001). At follow-up, the prevalences of global and severe abdominal obesity were significantly higher among women (both p values < 0.001, see Table 1). Moreover, the development of severe abdominal obesity (p < 0.001) was more common in women. The prevalence of common mental disorder was more frequent in women (all p < 0.001) at baseline and at the re-survey studies.

**Table 1:** Study participants’ characteristics at baseline (1984) and re-survey (1991) according to gender.

	Men		Women	
	Baseline (1984)	Re-survey (1991)	Baseline (1984)	Re-survey (1991)
n	1 488	1 488	1 900	1 900
**Age**, years	45.7 (15.7)	—-	44.6 (14.9)	—-
**Alcohol consumption**, units/day* ^†^	11 (4–24)	—-	4 (1–8)	—-
**Current tobacco smoking**, %	42.4		31.0	—-
**Marital status**, %				
Married or cohabitating	76.3	—-	74.7	—-
Single	23.7	—-	25.3	—-
**Socio-economic status**, %				
Non manual workers	48.1	—-	44.6	—-
Manual workers	51.9	—-	55.4	—-
**Physical activity level**, min/week^†^	210 (105–390)	—-	120 (60–210)	—-
**BMI**, kg/m^2^	25.0 (3.5)	25.7 (3.7)	24.4 (4.2)	25.4 (4.6)
**General obesity (BMI ≥ 30 kg/m** ^**2**^ **)**, %	7.4	10.4	10.0	14.0
**General obesity at resurvey (BMI ≥ 30)**, %**	—-	5.2	—-	6.4
**Waist circumference**, cm***	90.0 (10.1)	93.9 (10.6)	76.5 (10.6)	81.3 (11.4)
**Severe abdominal obesity (WC > 88/102 cm)**, %	11.2	19.2	13.3	24.4
**Severe abdominal obesity at re-survey (WC > 88/102), %#**	—-	4.3	—-	9.3
**GHQ score** ^†^	1 (0–4)	1 (0–5)	2 (0–6)	1 (0–6)
**Common mental disorder, %**	23.9	25.1	30.1	30.3
**Common mental disorder at re-survey 4, %@**	—-	17.4	—-	21.1
**Prescribed psychotropic** ^**‡**^ **drugs, %**	—-	2.0	—-	4.0

Data are mean (standard deviation) or percentages, unless specified.

*Among drinkers only, 1 248 women and 1 216 men.

** Among the 3 007 subjects with BMI < 30 kg/m^2^ at baseline.

# Among the 2 333 subjects with waist < 88 (women) 102 (men) cm at baseline.

@ Among the 2 461 subjects with GHQ score < = 4 at baseline.

***2 250 subjects for which data are available.

† Median values (25^th^— 75^th^ percentiles).

‡ The drugs prescribed include anti-depressants and anxiolytics.

### Baseline common mental disorders as a risk factor for re-survey global and abdominal obesity

In Table 2 we show the relation between common mental disorder at baseline and the development of general and abdominal obesity at the re-survey. Relative to those who were free of common mental disorder, men with the condition experienced a doubling of the risk of developing general obesity by re-survey; there was essentially no suggestion of an effect for global overweight. There was no relation between common mental disorder and waist circumference in men. In women, there was an association of common mental disorder at baseline with later overweight (p = 0.02). Also, the odd of developing common mental disorder was higher in women having baseline moderate (p = 0.001) or severe (p = 0.01) abdominal obesity compared to those with normal waist circumference. Throughout these analyses, controlling for a range of covariates had essentially no impact on the effect estimates.

**Table 2:** Odds ratios and 95% CIs for the relation of between common mental disorder among non-obese subjects at baseline (1984) and the development of global or abdominal obesity at re-survey (1991).

	Men				Women	
	BMI categories at re-survey				BMI categories at re-survey	
	18.5–24.9	25–29.9	> 30	18.5–24.9	25–29.9	> 30
**Baseline GHQ score**						
≤ 4	510	484	45	737	374	73
> 4	150	144	27	273	182	37
**Model 1**						
Common mental disorder	1.00 (ref)	1.02 (0.79–1.34)	2.03 (1.22–3.38)	1.00 (ref)	1.35 (1.08–1.69)	1.32 (0.86–2.01)
P for difference		0.83	0.007		0.009	0.20
**Model 2**						
Common mental disorder	1.00 (ref)	1.05 (0.81–1.38)	2.10 (1.23–3.55)	1.00 (ref)	1.30 (1.03–1.64)	1.26 (0.82–1.94)
P for difference		0.70	0.006		0.02	0.29
	**WC categories at re-survey**			**WC categories at re-survey**		
	**< 94**	**94–102**	**> 102**	**< 80**	**80–88**	**> 88**
**Baseline GHQ score**						
≤ 4	624	263	113	741	240	165
> 4	196	82	35	266	131	86
**Model 1**						
Common mental disorder	1.00 (ref)	1.01 (0.75–1.37)	1.01 (0.67–1.55)	1.00 (ref)	1.61 (1.25–2.09)	1.54 (1.14–2.08)
P for difference		0.92	0.93		0.001	0.005
**Model 2**						
Common mental disorder	1.00 (ref)	1.05 (0.77–1.43)	1.02 (0.66–1.56)	1.00 (ref)	1.57 (1.21–2.04)	1.48 (1.09–2.01)
P for difference		0.74	0.94		0.001	0.01

GHQ: 30 items General health questionnaire. BMI: body mass index. WC: waist circumference.

Multinomial logistic regression models were used with subjects having a body mass index between 18.5 and 24.9 kg/m² (both gender) or a waist circumference < 94 cm (men) /80 cm (women) being the reference.

Model 1: Adjusted for baseline age

Model 2: adjusted for baseline age, marital status, socio-economic status, alcohol consumption, tobacco smoking and physical activity.

In Table 3 we present the relation of changes in common mental disorder status between baseline and re-survey on the development of general or abdominal obesity at re-survey. Compared to study members free of common mental disorder at both surveys, men with common mental disorder at baseline and with normal mental health at re-survey had higher risk of obesity but not of overweight. The prevalence of neither moderate nor severe abdominal obesity in men differed across the different classes of exposition to common mental disorder. The prevalences of overweight, obesity and abdominal obesity were 61% to 200% significantly higher in women with common mental disorder at both surveys in comparison to those with normal mental health at both surveys. For both genders, these differences persisted when all the other studied covariates were included in the models.

**Table 3:** Odds ratios and 95% CIs for the relation between changes in common mental disorder status between baseline and re-survey on the development of general or abdominal obesity at re-survey.

Evolution of GHQ score betweenbaseline and re-survey	Men			Women	
	BMI categories at the re-survey study			BMI categories at the re-survey study	
	25–29.9	> 30		25–29.9	> 30
**Model 1**					
Normal mental health at both surveys	1.00 (ref)	1.00 (ref)		1.00 (ref)	1.00 (ref)
Common mental disorder at baseline only	1.02 (0.79–1.34)	2.81 (1.52–5.22)		0.99 (0.72–1.35)	1.19 (0.66–2.15)
Common mental disorder at re-survey only	0.97 (0.70–1.35)	1.15 (0.53–2.47)		0.84 (0.61–1.15)	1.62 (0.95–2.76)
Common mental disorder at both surveys	1.02 (0.72–1.46)	1.37 (0.63–2.96)		1.67 (1.24–2.24)	1.83 (1.06–3.17)
P for difference	0.99	0.01		0.001	0.09
**Model 2**					
Normal mental health at both surveys	1.00 (ref)	1.00 (ref)		1.00 (ref)	1.00 (ref)
Common mental disorder at baseline only	1.04 (0.73–1.51)	3.03 (1.60–5.76)		0.96 (0.70–1.31)	1.19 (0.65–2.16)
Common mental disorder at re-survey only	0.98 (0.70–1.37)	1.11 (0.51–2.42)		0.85 (0.62–1.17)	1.79 (1.04–3.07)
Common mental disorder at both surveys	1.05 (0.73–1.51)	1.36 (0.62–2.99)		1.61 (1.20–2.17)	1.77 (1.02–3.08)
P for difference	0.98	0.007		0.004	0.08
	**WC categories at the re-survey study**			**WC categories at the re-survey study**	
	**94–102**		**> 102**	**80–88**	**> 88**
**Model 1**					
Normal mental health at both surveys	1.00 (ref)		1.00 (ref)	1.00 (ref)	1.00 (ref)
Common mental disorder at baseline only	1.15 (0.78–1.70)		1.27 (0.73–1.70)	1.33 (0.93–1.88)	1.34 (0.88–2.04)
Common mental disorder at re-survey only	0.67 (0.44–1.01)		1.35 (0.83–2.21)	0.84 (0.58–1.23)	1.41 (0.95–2.08)
Common mental disorder at both surveys	0.78 (0.52–1.18)		0.92 (0.52–1.65)	1.82 (1.30–2.55)	2.03 (1.38–2.99)
P for difference	0.13		0.54	0.001	0.003
**Model 2**					
Normal mental health at both surveys	1.00 (ref)		1.00 (ref)	1.00 (ref)	1.00 (ref)
Common mental disorder at baseline only	1.20 (0.80–1.80)		1.27 (0.72–2.23)	1.33 (0.93–1.89)	1.29 (0.84–1.97)
Common mental disorder at re-survey only	0.67 (0.44–1.02)		1.22 (0.74–2.02)	0.83 (0.56–1.21)	1.40 (0.94–20.8)
Common mental disorder at both surveys	0.81 (0.53–1.24)		0.89 (0.50–1.61)	1.72 (1.22–2.41)	1.94 (1.31–2.86)
P for difference	0.13		0.69	0.003	0.008

GHQ: 30 items General health questionnaire. BMI: body mass index. WC: waist circumference.

Multinomial logistic regression models were used with subjects having a GHQ score ≤ 4 both at baseline and at the re-survey study, and with a BMI between 18.5 and 24.9 kg/m² (both gender) or a WC < 94 cm (men) /80 cm (women) being the reference.

Model 1: Adjusted for baseline age.

Model 2: adjusted for baseline age, marital status, socio-economic status, alcohol consumption, tobacco smoking and physical activity.

The sample sizes are 1344 and 1703 for men and women respectively when BMI is used to characterize obesity, and 1269 and 1662 for men and women respectively when WC is used to characterize obesity.

### General and abdominal obesity as a risk factor for the development of common mental disorders at re-survey

In Table 4 are presented the relationship between general and abdominal obesity at baseline and common mental disorder at re-survey. Neither baseline BMI categories nor baseline waist circumference categories was associated with common mental disorder at re-survey in either women or men. Compared to women having a BMI < 30 kg/m^2^ at both surveys (data not shown), women having a BMI > 30 kg/m^2^ at baseline but < 30 kg/m^2^ at re-survey study had higher risk of common mental disorder when adjustments were made for age (1.74, 1.04–2.91) or in a fully-adjusted model (1.81, 1.07–2.09).

**Table 4:** Odds ratios and 95% CIs for the relation between general or abdominal obesity among subjects free of common mental disorders at baseline (1984) and the development of common mental disorders at the re-survey study (1991).

	Common mental disorder (GHQ score > 4) at the re-survey study	
	Men	Women
**Baseline BMI categories (kg/m** ^**2**^ **)**		
18.5 ≤ BMI < 25	114/611	170/814
25 ≤ BMI < 30	71/427	68/348
BMI **≥** 30	11/84	29/118
**Model 1**		
18.5 ≤ BMI < 25	1.00 (ref)	1.00 (ref)
25 ≤ BMI < 30	0.90 (0.64–1.26)	0.90 (0.66–1.24)
BMI **≥** 30	0.68 (0.35–1.32)	1.21 (0.46–1.90)
P for difference	0.49	0.51
**Model 2**		
18.5 ≤ BMI < 25	1.00 (ref)	1.00 (ref)
25 ≤ BMI < 30	0.94 (0.66–1.32)	0.93 (0.68–1.29)
BMI **≥** 30	0.70 (0.36–1.38)	1.33 (0.84–2.12)
P for difference	0.59	0.38
**Baseline WC categories (cm)**		
WC ≤ 80/94	132/771	201/936
80/94 < WC ≤ 88/102	43/230	38/212
WC > 88/102	22/127	40/167
**Model 1**		
WC ≤ 80/94	1.00 (ref)	1.00 (ref)
80/94 < WC ≤ 88/102	1.18 (0.80–1.75)	0.77 (0.52–1.14)
WC > 88/102	1.10 (0.66–1.84)	1.11 (0.74–1.65)
P for difference	0.70	0.32
**Model 2**		
WC ≤ 80/94	1.00 (ref)	1.00 (ref)
80/94 < WC ≤ 88/102	1.16 (0.78–1.73)	0.78 (0.53–1.16)
WC > 88/102	1.11 (0.66–1.87)	1.18 (0.79–1.78)
P for difference	0.75	0.27

GHQ: 30 items General health questionnaire. BMI: body mass index. WC: waist circumference.

Model 1: Adjusted for baseline age

Model 2: adjusted for baseline age, marital status, socio-economic status, alcohol consumption, tobacco smoking and physical activity.

### Sensitivity analyses

After adjusting for age and the other covariates, participants with baseline common mental disorder (GHQ score > 4) experienced greater increase in BMI (women: 0.44 + 0.12 kg/m^2^, p < 0.001 and men: 0.16 + 0.12 kg/m^2^, p = 0.19) and in WC (women: 1.08 + 0.39 cm, p = 0.005 and men: 0.34 + 0.39, p = 0.39) only in women. Age adjusted changes in GHQ score did not vary according to BMI or waist circumference categories in both genders.

## Discussion

Findings from this well characterized population-based cohort of British men and women provide evidence that common mental disorders may be a risk factor for future obesity. This relationship was found for measures of obesity based on BMI (men and women) and waist circumference (women only). Similar patterns of associations were found in women when BMI and waist circumference as a continuous, rather than categorical, variables were used as markers of anthropometry. We found no evidence of a bidirectional or reciprocal association in which obesity also predicts future risk of common mental disorder.

Despite high response at the successive data collection phases, differential drop out rates between the two surveys due to death, refusal and non-tracing affected the sample distribution. The loss to follow-up in the HALS is similar to other studies. This attrition has implications for estimating the prevalence of a given risk factor or disease. However, as we have also demonstrated with these data, using the exemplar of CVD risk, this attrition has essentially no impact on the estimation of risk factor-disease association. Thus, in relating risk factors to CVD mortality, the same magnitude of association was evident in people who participated in the baseline survey but not the resurvey relative to the group that took part in both the baseline survey and re-survey (p-value for interaction: 0.108).[26] Nevertheless, the distributions of the HALS-2 population compared reasonably well with that of the 1991 British census data respectively.[19,20,27] Common mental disorder was assessed using the self-administered 30-item GHQ, which focuses on self-reported symptoms of anxiety and depression, and associated psychosocial dysfunction.[21] This device is designed as a screening instrument for use in community settings. It has been validated against standardized clinical interviews and has shown high reliability.[28] However, the GHQ is not a measure of clinically recognised psychiatric disorder.[21] Although the symptom scale is reliable, we cannot be certain that our findings would be directly transferable to individuals meeting the DSM–IV[29]or ICD–10[30] criteria for specific mental disorders such as major depressive disorder or anxiety disorders.

Anti-depressive drugs intake was not recorded during the HALS-1 Study. This would have helped in better characterizing subjects at high risk of developing obesity by the time of the re-survey study. Anti-depressant use in the general population is rare as found during the re-survey study (2% of men and 4% of women). Few participants were present in the higher categories of obesity. This could lead to unstable findings. However, significant results in the presence of a low statistical power usually indicate a strong association.

Among previous studies that evaluated the association between mental health and obesity, only two used the GHQ to characterize common mental disorder.[17,31] Our findings confirm the results previously described [17,31] such as common mental disorder might be a risk factor of future general obesity. None of these studies examined the association with both general *and* abdominal adiposity. Other longitudinal studies have evaluated various markers of mental health in predicting general obesity, and found a positive[32,33] or no association.[8,9,34] Pan et al [16] reported a bidirectional association between depression and obesity in older women. Luppino et al [7] reported in the most recent meta-analysis of longitudinal studies in this field that baseline depression (symptoms and disorder) was predictive of general obesity over time (OR: 1.58, 1.33–1.87). The specific role of abdominal adiposity was not evaluated in this report.

In agreement with a previous study using repeat measures of GHQ to characterize mental health,[17] we found no robust association between obesity and subsequent common mental disorder. However, using various surrogates of mental health, others authors have found a positive [6,35] or no association.[8,9] It has been suggested that the association between common mental disorder and general obesity might be influenced by gender, mean age of study participants at baseline, follow-up duration and ethnic groups.[4,7,36] However, we performed statistical analyses separately for men and women, had little variation in follow-up periods between participants and the findings were robust to adjustment for age. This suggests that major bias is unlikely.

Although evidence of biological links between mental health and obesity remain complex and not definitive,[3,10,37] it seems relevant to highlight the most current hypotheses explaining the possibility of a biological pathway. First, common mental disorders are associated with eating disorders, which could influence future changes in adiposity.[38,39] Physical inactivity, a major contributing factor to obesity, is more prevalent among people with mental health problems.[40] Furthermore, commonly used pharmacological treatments for mental health problems have known side effects that may result in weight gain, weight loss or both.[13,41] The fact that mental health disorders induce an increase of weight over time may also be related to neuroendocrine disturbances, such as long-term activation of the hypothalamic–pituitary–adrenocortical (HPA) axis in the context of an increased visceral fat.[42] Cortisol, in the presence of insulin, inhibits lipid-mobilizing enzymes, a process mediated by glucocorticoid receptors that are found in fat depots and especially in intra-abdominal visceral fat.[43]

The association of obesity with future depression may be related to diagnosis of a chronic disease (or the treatment in terms of drugs and dietary restrictions) rather than to obesity. This possibility is consistent with our findings suggesting no real association between obesity and subsequent common mental disorder. Obesity can be seen as an inflammatory state, as weight gain has been shown to activate inflammatory pathways[44] and inflammation in turn has been associated with depression.[45]

In conclusion, our findings of a longitudinal association between common mental disorders and obesity may have important implications for clinical practice. Because weight gain appears to be a late consequence of common mental disorders, care providers should be aware that within patients showing symptoms of psychological distress or depression, weight should be monitored carefully. This awareness should lead to prevention, early detection, and co treatment of patients at risk, which could ultimately reduce the burden of common mental disorders. Longitudinal epidemiological research is especially warranted to further establish the plausible mechanisms underlying the link between common mental disorders and the onset of obesity, such medication use, physical activity and dietary patterns.

## Supporting Information

S1 Table Comparison of baseline characteristics of included and excluded participants. (DOC)
