# Correction: Is the Relationship between Common Mental Disorder and Adiposity Bidirectional? Prospective Analyses of a UK General Population-Based Study

**DOI:** 10.1371/journal.pone.0134197

**Published:** 2015-07-24

**Authors:** Léopold K. Fezeu, G. David Batty, Catharine R. Gale, Mika Kivimaki, Serge Hercberg, Sebastien Czernichow

The second author’s name is spelled incorrectly. The correct name is: G. David Batty. The correct citation is: Fezeu LK, Batty GD, Gale CR, Kivimaki M, Hercberg S, Czernichow S (2015) Is the Relationship between Common Mental Disorder and Adiposity Bidirectional? Prospective Analyses of a UK General Population-Based Study. PLoS ONE 10(5): e0119970. doi:10.1371/journal.pone.0119970

The affiliations for the second and third authors are incorrect. G. David Batty is not affiliated with #2, but with #3: Department of Epidemiology & Public Health, University College London, London, United Kingdom. Catherine R. Gale is not affiliated with #4 but with #9 and #10: MRC Lifecourse Epidemiology Unit, University of Southampton, Southampton, UK and Centre for Cognitive Ageing and Cognitive Epidemiology, Department of Psychology, University of Edinburgh, UK.
