# Supplementary material for: Is the Relationship between Common Mental Disorder and Adiposity Bidirectional? Prospective Analyses of a UK General Population-Based Study
Source: PLoS One. 2015 May 18;10(5):e0119970. doi: 10.1371/journal.pone.0119970 (PMC4436271; doi:10.1371/journal.pone.0119970)
Supplement: S1 Table — (DOC) [file pone.0119970.s001.doc]

**S1** T**able:** Comparison of baseline characteristics of included and excluded participants

|  |  | **Included** |  | **Excluded** |  | **P** |
| --- | --- | --- | --- | --- | --- | --- |
| **N** |  | **3388** |  | **3375** |  |  |
| **Men, %** |  | 43.0 |  | 43.9 |  | 0.42 |
| **Age**, years |  | 45.1 + 15.3 |  | 46.3 + 19.0 |  | 0.002 |
| **Alcohol consumption**, units/day |  | 6 (2 – 14) |  | 7 (2 – 16) |  | 0.39 |
| **Current tobacco smoking**, % |  | 35.4 |  | 43.3 |  | 0.001 |
| **Marital status**, % |  |  |  |  |  | 0.001 |
| Married or cohabitating |  | 90.7 |  | 86.0 |  |  |
| Single |  | 9.3 |  | 14.0 |  |  |
| **Occupational social class**, % |  |  |  |  |  | 0.001 |
| Non-manual |  | 46.5 |  | 40.5 |  |  |
| Manual |  | 53.5 |  | 59.5 |  |  |
| **BMI**, kg/m^2^ |  | 24.6 + 3.9 |  | 24.5 + 4.2 |  | 0.64 |
| **General obesity (BMI ≥ 30 kg/m^2^)**, % |  | 8.7 |  | 9.7 |  | 0.27 |
| **Waist circumference**, cm |  | 82.5 + 12.4 |  | 83.4 + 12.9 |  | 0.004 |
| **Severe abdominal obesity (WC > 88/102 cm)**, % |  | 12.8 |  | 15.9 |  | 0.001 |
| **GHQ score** |  | 1 (0 – 4) |  | 1 (0 – 5) |  | 0.32 |

† Median values (25^th^ – 75^th^ percentiles).
